# Virtual Screening of FDA-Approved Drugs against Triose Phosphate Isomerase from *Entamoeba histolytica* and *Giardia lamblia* Identifies Inhibitors of Their Trophozoite Growth Phase

**DOI:** 10.3390/ijms22115943

**Published:** 2021-05-31

**Authors:** Alfredo Juárez-Saldivar, Elizabeth Barbosa-Cabrera, Edgar E. Lara-Ramírez, Alma D. Paz-González, Ana V. Martínez-Vázquez, Virgilio Bocanegra-García, Isidro Palos, Nuria E. Campillo, Gildardo Rivera

**Affiliations:** 1 Laboratorio de Biotecnología Farmacéutica, Centro de Biotecnología Genómica, Instituto Politécnico Nacional, Reynosa 88710, Mexico; ajuarezs1500@gmail.com (A.J.-S.); delpaz11@gmail.com (A.D.P.-G.); 2 Sección de Estudios de Posgrado e Investigación, Escuela Superior de Medicina, Instituto Politécnico Nacional, Ciudad de México 11340, Mexico; rebc78@yahoo.com.mx; 3 Unidad de Investigación Biomédica de Zacatecas, Instituto Mexicano Del Seguro Social, Zacatecas 98617, Mexico; elarar0700@hotmail.com; 4 Centro de Biotecnología Genómica, Instituto Politécnico Nacional, Reynosa 88710, Mexico; avmartinez07@gmail.com (A.V.M.-V.); vbocanegg@yahoo.com (V.B.-G.); 5 Unidad Académica Multidisciplinaria Reynosa-Rodhe, Universidad Autónoma Tamaulipas, Carr. Reynosa-San Fernando, Reynosa 88779, Mexico; isi_palos@hotmail.com; 6 Centro de Investigaciones Biológicas Margarita Salas (CIB-CSIC), Ramiro de Maeztu 9, 28040 Madrid, Spain; nuria.campillo@csic.es

**Keywords:** protozoa, FDA, virtual screening, drug repositioning, molecular docking

## Abstract

Infectious diseases caused by intestinal protozoan, such as *Entamoeba histolytica* (*E. histolytica*) and *Giardia lamblia* (*G. lamblia*) are a worldwide public health issue. They affect more than 70 million people every year. They colonize intestines causing primarily diarrhea; nevertheless, these infections can lead to more serious complications. The treatment of choice, metronidazole, is in doubt due to adverse effects and resistance. Therefore, there is a need for new compounds against these parasites. In this work, a structure-based virtual screening of FDA-approved drugs was performed to identify compounds with antiprotozoal activity. The glycolytic enzyme triosephosphate isomerase, present in both *E. histolytica* and *G. lamblia*, was used as the drug target. The compounds with the best average docking score on both structures were selected for the in vitro evaluation. Three compounds, chlorhexidine, tolcapone, and imatinib, were capable of inhibit growth on *G. lamblia* trophozoites (0.05–4.935 μg/mL), while folic acid showed activity against *E. histolytica* (0.186 μg/mL) and *G. lamblia* (5.342 μg/mL).

## 1. Introduction

Intestinal protozoa are eukaryotic unicellular organisms that cause several diseases to humans and animals. Mainly, they affect developing regions; nevertheless, due to globalization and the increase of human migration, some of these diseases are becoming a health threat all over the world [1]. The parasites, *Entamoeba histolytica* (*E. histolytica*) and *Giardia lamblia* (*G. lamblia*), are the major agents causing parasitosis affecting more than 70 million people every year [2,3]. *E. histolytica* causes amoebiasis, characterized by pyrexia, abdominal cramping and dysentery symptoms. This parasite can migrate to other organs, like liver, causing amebic liver abscess [4,5]. *G. lamblia* colonizes small intestine, causing giardiasis disease, one of the most common causes of diarrhea in children and adults [6,7]. Other symptoms caused by *G. lamblia* infection are greasy stool, flatulence and bloating [8]. Some reports indicate up to 10% of coinfection with both parasites in migrant populations and returning travelers [9,10].

The main line of treatment against these parasites is metronidazole. It has been used for over 60 years, but the increase of drug resistance leads to higher dose use in treatment, and therefore more severe side effects [8]. Consequently, there is a need for new and safe alternatives for amoebiasis and giardiasis treatment. One of the main strategies used in the finding of active compounds is drug repositioning, which reduces cost and time in drug [11,12]. The increment of free available biological data and advances in computational techniques have led to several new ways of virtual screening, that, compared to in vitro evaluation, have become a cheaper and faster alternative to screen drug libraries. Thus, ligand-based and structure-based virtual drug repositioning are widely used today [13,14,15,16,17].

Structure-based methods involve structural information of enzymes and/or other types of proteins used as drug targets. In the last decade, new drug targets have been studied in the search of compounds against intestinal protozoa [18,19,20,21,22,23]. Among these drug targets, triosephosphate isomerase (TIM), a glycolytic enzyme, has been as widely explored on many species; nevertheless, studies on *E. histolytica* and *G. lamblia* lack depth [24,25,26]. Recently, new series of compounds have been reported to inhibit TIM from *E. histolytica* (TIM*Eh*) and TIM from *G. lamblia* (TIM*Gl*) [27,28]. Therefore, in this work, known inhibitor compounds were used to identify structural characteristics and main interactions involved in the binding to TIM*Eh* and TIM*Gl*. Later, a virtual screen of library of 1466 FDA-approved drugs was performed on this drug target to identify new antiprotozoal drugs with possible broad-spectrum activity.

## 2. Results and Discussion

### 2.1. Docking-Based Virtual Screening on TIMEh and TIMGl

**Figure 1:**
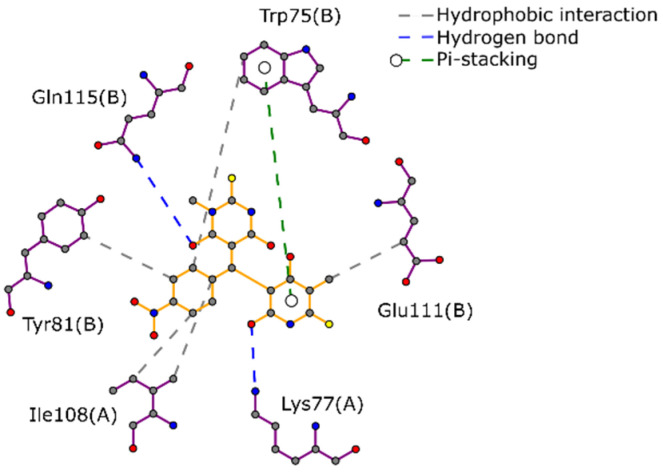
2D interaction diagram between compound D4, an inhibitor, and TIM*Eh* on the interface.

Before starting the screening virtual (SV) of the FDA library against TIM*Eh*, the docking of a known inhibitor, compound D4, denominates 5,5′-[(4-nitrophenyl)methylene]bis-6-hydroxy-2-mercapto-3-methyl-4(3H)-pyrimidinone)[27], was used both to validate the docking protocol and to use this compound as a control compound. The docking score obtained for D4 was -5.4 kcal/mol. The non-covalent interactions between TIM*Eh* and D4 were calculated using PLIP. As Figure 1 shows, the main interactions were hydrophobic with residues Trp75(B), Tyr81(B), Ile108(A) and Glu111(B). The residue Trp75(B) also had a π-stacking interaction. This compound also formed two hydrogen bonds with Lys77(A) and Gln115(B). 

Once the D4 inhibitor was studied, 1466 FDA drugs were docked against TIM*Eh*. The compounds were ranked based on their docking score, and the top ten are described in Table S1. These ten compounds contain several aromatic rings (like D4); therefore, hydrophobics and π-stacking interactions were expected (Table S2). Risperidone and iloperidone are antipsychotic drugs with a similar structure composed of a benzoxazole attached to a fluorine atom and a piperidine ring. On the other hand, folic acid, rilpivirine and pralatrexate, used as chemotherapy drugs, share a benzaldehyde and a glutamic acid in their structures. Interesting, bisacodyl is a tricyclic compound like D4, which also contains two methyl acetate that could form a hydrogen bond. According to the interaction profiles calculated with PLIP (Table S2), the most common interactions between TIM*Eh* and these structures were π-stacking Trp75, a hydrogen bond with Gln115 and hydrophobic interaction with Ile108.

**Figure 2:**
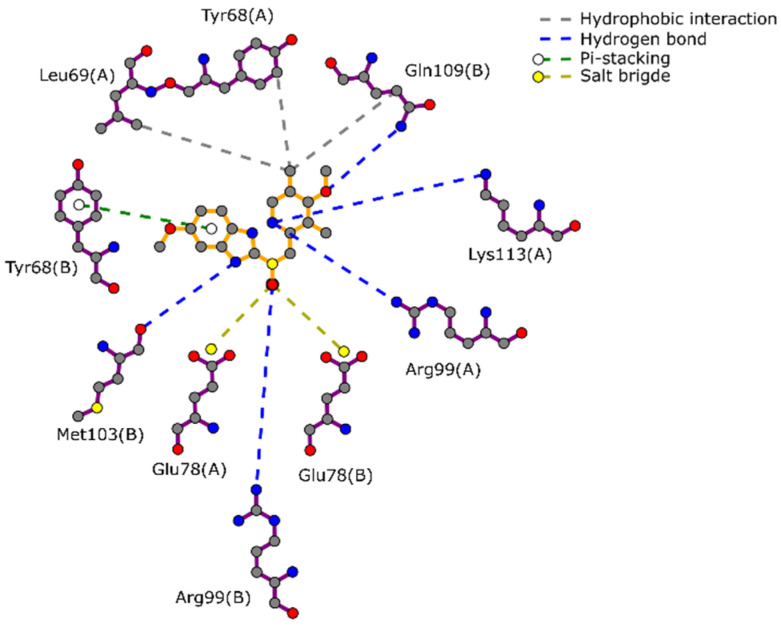
2D interaction diagram between omeprazole drug and TIM*Gl* on the interface.

In the case of TIM*Gl*, the omeprazole drug has been previously reported as an effective inhibitor [28]. Hence, omeprazole was docked at the interface to identify the possible interactions involved in its binding to TIM*Gl*. The docking score for omeprazol was −7.7 kcal/mol and its interactions are shown in Figure 2. In this diagram, several hydrogen bonds with Arg99(A), Arg99(B), Met103(B), Gln109(B) and Lys113(A) are indicated, as well as some hydrophobic interactions with Tyr68(A), Leu69(A) and Gln109(B). A salt bridge could be formed with Glu78 from each monomer. Also, Tyr68(B) interacts through π-stacking with the benzimidazole moiety.

For the 1466 FDA-approved drugs virtual screening on TIM*Gl*, the top ten are outlined in Table S3. These compounds are structurally diverse with the exception of sulfasalazine and eltrombopag that share a benzoic acid and an aniline ring. Nevertheless, their interaction profile showed some similar interactions (Table S4). The most common interactions are the π-stacking interaction with Tyr68, and hydrogen bonding with Arg99, Gln109 andLys113, which are shared with the known inhibitor.

In general, binding energies on TIM*Gl* were better than for TIM*Eh* and there were no compounds ranked within the top ten of both species. Therefore, docking scores of each compound were merged and obtain the average docking score on both TIM*Eh* and TIM*Gl* and thus identify compounds with potential broad effect. Compounds were re-ranked based on this average score and presented in Table S5. There are some structural characteristics to point out. Previously reported TIM inhibitors bound to the interface by aromatic interactions [29]. Interestingly, re-ranked compounds have several aromatic rings in their structure which could bind through π-stacking or π-cation interactions. Among these compounds, chlorhexidine is the only one which structure contains halogen atoms, and also has several H-bond donors, which are one of the main characteristics in omeprazole binding to TIM*Gl*. Pemetrexed and folic acid have a similar structure, they contain a glutamic acid group attached to a benzoic acid. The difference is that pemetrexed has pyrrole[2,3-d]pyrimidine, folic acid has pteridine ring; nevertheless, both functional groups are suitable for aromatic interaction and hydrogen bonding. Pyrimidine structure is also present in imatinib structure, along with a benzoic acid. On the other hand, dolasetron have an indol group, similar to the pyrrolepyrimidine in folic acid. Tolcapone and arbutamine have another interesting group, which is benzenediol. These structures showed the common functional groups that could be explore in the early drug design steps process to develop new TIM inhibitors.

### 2.2. In Vitro Activity

Due to the fact that TIM is an essential protein for parasite survival, inhibition of TIM would inhibit cellular growth. On this basis, we considered that compounds selected from the virtual screening would have an inhibitory effect on both *E. histolytica* and *G. lamblia*. In order to explore a variety of chemical structures against intestinal protozoa, based on the in silico results, chlorhexidine, tolcapone, imatinib and folic acid were selected for their evaluation in vitro. Table 1 shows that these compounds were capable of inhibiting *G. lamblia* growth and only folic acid has inhibitory activity against both parasites. Only two of these compounds, chlorhexidine and imatinib, have previous report of activity against *G. lamblia* [30]. Selected compounds have a better in vitro activity on *G. lamblia*; only folic acid showed inhibitory activity on *E. histolytica*.

**Figure 3:**
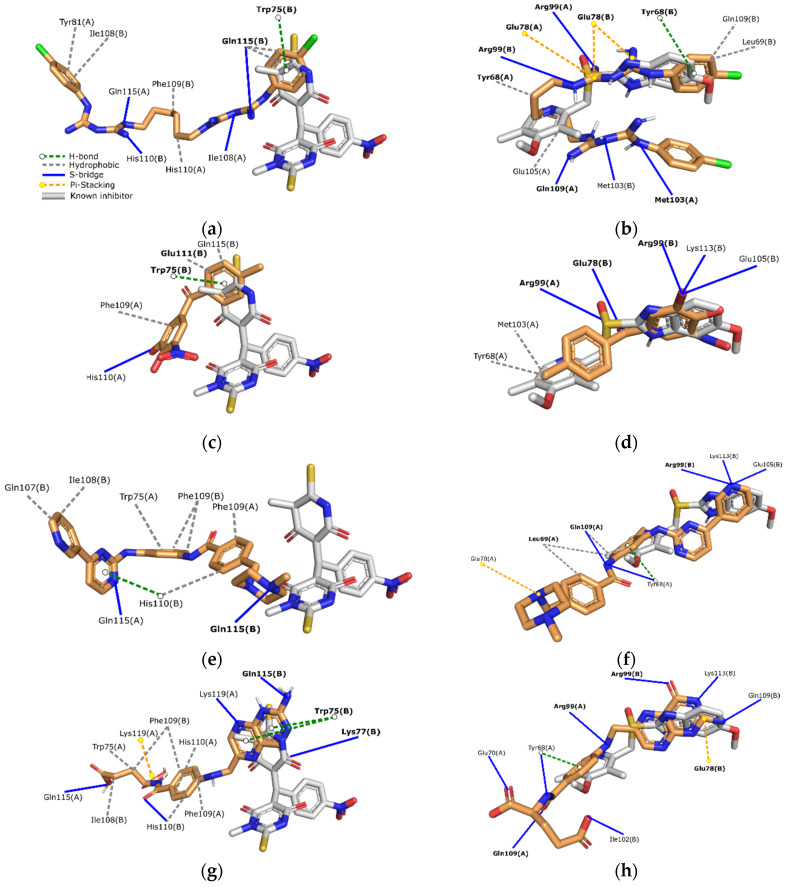
Interaction diagram of selected compound in complex: (**a**) Chlorhexidine on TIM*Eh*; (**b**) Chlorhexidine on TIM*Gl*; (**c**) Tolcapone on TIM*Eh*; (**d**) Tolcapone on TIM*Gl*; (**e**) Imatinib on TIM*Eh*; (**f**) Imatinib on TIM*Gl*; (**g**) Folic acid on TIM*Eh*; (**h**) Folic acid on TIM*Gl*. Residues in bold text represent interactions shared with the known inhibitor.

Chlorhexidine is an antiseptic compound used to treat bacterial and fungal infection. Its structure is composed of two chlorophenyl guanide groups linked by a hexamethylene bridge. As Figure 3 shows, this structure has a higher number of interactions in TIM*Gl* than TIM*Eh*, which explains the differences on docking score. The high number of hydrogen bonds represent an interesting feature that is directly related to binding stability along with the π-stacking interactions with Tyr68B (TIM*Gl*).

Tolcapone is a benzophenone used in the treatment of Parkinson disease. This structure has a few interactions with TIM*Eh* but several hydrogen bonds with TIM*Gl*. These interactions could explain why tolcapone was only active against *G. lamblia*.

In the case of imatinib, an anticancer drug, interactions are mainly by hydrogen bonding and hydrophobic interactions despite the four rings in its structure which commonly form π-stacking interactions with aromatic residues.

Interestingly, folic acid, a supplement, showed a high number and diverse types of interactions in both TIM*Eh* and TIM*Gl*. This was the only evaluated compound that showed inhibitory activity against *E. histolytica* and *G. lamblia*, there are some reports considering the impact of folic acid in parasitic infections, but no conclusion is given about its use as an antiprotozoal compound [31,32]. Its interaction diagram summarized its potential binding mechanism in both protozoal TIM. It mainly interacts by hydrogen bonding. Also, it forms a π-stacking interaction with Trp75B in TIM*Eh* and by π-cation with Arg99 on TIM*Gl*. Nevertheless, specific inhibition studies on TIM*Eh* and TIM*Gl* along with more robust computational analysis are needed to validate these findings.

## 3. Materials and Methods

### 3.1. Docking-Based Virtual Screening

Crystal structures of TIM proteins of *E. histolytica* and *G. lamblia* (PDB IDs, TIM*Eh*: 1M6J and TIM*Gl*: 4BI7) were retrieved from Protein Data Bank (PDB, RCSB PDB: Homepage accessed on 8 October 2020). Each structure was prepared with the dock prep tool from USFC Chimera software (University of California, San Francisco, CA, USA) [33]. In this step, all ions, water molecules and co-crystalized ligands were removed, and missing side chains were added. TIM is a dimeric structure composed of a TIM-barrel structure in each monomer with the catalytic site being inside this barrel. Due to the high similarity of the catalytic site among all TIM structures, species-specific inactivation is focused on the dimeric interface. Therefore, conformational search space for docking was defined as a 20 × 20 × 20 Å box centered in PDB 1M6J at x = 14.620, y = 27.866 and z = 14.108, and in PDB 4BI7 at x = 5.710, y = −0.023 and z = −28.240. Later, a total of 2454 SDF structures corresponding to FDA-approved drugs were retrieved from DrugBank (DrugBank|Pharmaceutical Knowledge Base|API Integrations accessed on 8 October 2020) [34]. These structures, along with D4 and omeprazole (inhibitors used as control for TIM*Eh* and TIM*Gl*, respectively), were split, minimized and converted to Mol2 format using open babel [35]. Only those with a molecular weight between 100 and 900 Da were used in the subsequent steps. A total of 1466 was successfully prepared for docking. The rest were discarded due to its molecular weight, minimizing errors and non-supported atom types. Additionally, AutoDockTools (ADT/AutoDockTools—AutoDock(scripps.edu) accessed on 8 October 2020) [36] was used to specify Gasteiger partial charges and AutoDock atom types to ligands and receptors. Then, docking was performed by Autodock vina (vina) [37] and in-house python scripts to automate calculations (Figure S1). Finally, the non-covalent interactions of the docked complexes were calculated with PLIP (protein–ligand interaction profiler) [38].

### 3.2. In Vitro Activity

*E. histolytica* strain HM1-IMSS was grown in TYI-S-33 medium supplemented with 10% heat-inactivated bovine serum (Sigma Adrich, Toluca, Mexico). *G. lamblia* strain IMSS:8909:1 trophozoites used in all experiments were cultivated axenically at 37 °C in TYI-S-33 modified medium supplemented with 10% calf serum and bovine bile. In vitro susceptibility tests were performed using *E. histolytica* (6 × 10^3^) or *G. lamblia* (5 × 10^4^) trophozoites were incubated for 48 h at 37 °C in the presence of different concentrations (2.5–200 μg/mL) of pure compounds in DMSO at 2%. After incubation, the trophozoites were detached by chilling and 50 μL samples of each tube were subcultured in fresh medium for another 24 h. The final number of parasites was determined in Neubauer. Then, data were analyzed using probit analysis. The percentage of trophozoites surviving was calculated by comparison with the growth in the control group. The plot of probit against log concentration was made, the best straight line was determined by regression analysis, and the IC_50_ values were calculated. The regression coefficient, its level of significance (*p* < 0.05 indicates significant difference between groups), and correlation coefficient were calculated and 95% confidence interval (CI) values determined. ADMET characteristics of selected compounds are shown in Table S3 [34].

## 4. Conclusions

In this work, virtual screening based on molecular docking was used to identify potential antiprotozoal compound among FDA-approved drugs that binds to the TIM from *E. histolytica* and *G. lamblia*. Those with a lowest average docking score were selected for in vitro evaluation. Four compounds, chlorhexidine, tolcapone, imatinib and folic acid, were capable of inhibiting growth of trophozoite of *G. lamblia* with an IC_50_ below standard treatment. Folic acid also showed activity against *E. histolytica*. Although these results are promising, more studies are needed to understand the mechanism underlying the inhibitory activity of these compounds.

## Figures and Tables

**Table 1:** Biological activity of four FDA drugs against trophozoites from *E. histolytica* and *G. lamblia*.

Compound.	IC_50_ *E. histolytica* (μg/mL)	IC_50_ *G. lamblia* (μg/mL)
Metronidazole	0.205	7.8
D4	8.306 ± 1.616 ^1^	-
Omeprazol	-	0.025 ^2^
Chlorhexidine	> 100	4.93 ± 0.005
Tolcapone	> 100	0.05 ± 0.002
Imatinib	> 100	3.46 ± 0.005
Folic acid	0.186 ± 0.003	5.34 ± 0.007

^1^ [26], ^2^ [28], Half-maximal inhibitory concentration (IC_50_).

## Supplementary Materials

The following are available online at https://www.mdpi.com/article/10.3390/ijms22115943/s1.
