# Supplementary material for: Virtual Screening of FDA-Approved Drugs against Triose Phosphate Isomerase from Entamoeba histolytica and Giardia lamblia Identifies Inhibitors of Their Trophozoite Growth Phase
Source: Int J Mol Sci. 2021 May 31;22(11):5943. doi: 10.3390/ijms22115943 (PMC8198924; doi:10.3390/ijms22115943)
Supplement: Supplementary file 1 [file ijms-22-05943-s001.zip › ijms-1216642-supplementary.pdf]

**Table S1.** Top ten FDA drugs from virtual screening on active site of TIM*Eh*.

| **Compound** | **Structure** | **Docking score (Kcal/mol)** | **Description** |
|---|---|---|---|
| Risperidone | 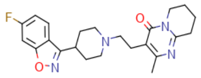 | -7.5 | Antipsychotic. |
| Pemetrexed | 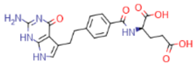 | -7.0 | Chemotherapy. |
| Folic Acid | 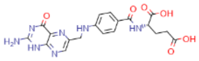 | -6.9 | Supplementation agent. |
| Chlorhexidine | 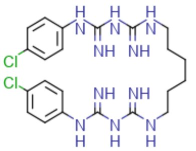 | -6.8 | Broad-spectrum antimicrobial. |
| Isocarboxazid | 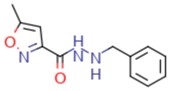 | -6.8 | Treatment of major depression. |
| Rilpivirine | 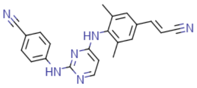 | -6.8 | Treatment of HIV-1 infections. |
| Iloperidone | 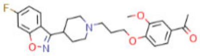 | -6.7 | Antipsychotic. |
| Pralatrexate | 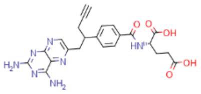 | -6.7 | Treatment of relapsed or refractory peripheral T-cell lymphoma. |
| Glycerol phenylbutyrate | 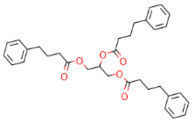 | -6.7 | Therapy for chronic management of patients with urea cycle disorders. |
| Bisacodyl | 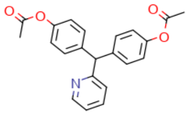 | -6.7 | Prescribed for constipation and neurogenic bowel dysfunction. |

**Table S2.** Interaction profile of top ten compound from virtual screening on TIM*Eh*.

| **Compound** | **Interactions** | | |
|---|---|---|---|
| Risperidone | 1 x hphobic Trp75(B) | 1 x hphobic His110(B) | 1 x hbond Gln115(B) |
| | 1 x hphobic Ile108(A) | 2 x hphobic Gln115(B) | 1 x pistack His110(A) |
| Pemetrexed | 1 x hphobic Trp75(A) | 1 x hbond Lys77(B) | 1 x hbond Lys119(B) |
| | 1 x hphobic Ile108(B) | 1 x hbond His110(B) | 1 x sbridge His110(B) |
| | 2 x hphobic Phe109(A) | 1 x hbond Glu111(B) | 1 x sbridge Lys119(A) |

| | | | |
|---|---|---|---|
| | 1 x hphobic Phe109(B) | 1 x hbond Gln115(A) | 2 x pistack Trp75(B) |
| | 1 x hphobic His110(B) | 2 x hbond Gln115(B) | |
| Folic Acid | 1 x hphobic Phe109(B) | 1 x hbond Phe109(B) | 2 x hbond Gln115(B) |
| | 1 x hphobic His110(A) | 1 x hbond His110(A) | 1 x sbridge His110(B) |
| | 1 x hbond Lys77(B) | 1 x hbond His110(B) | 1 x pistack Trp75(B) |
| | 1 x hbond Ile108(B) | 1 x hbond Gln115(A) | |
| Chlorhexidine | 1 x hphobic Tyr81(A) | 2 x hphobic Gln115(B) | 1 x hbond Gln115(A) |
| | 1 x hphobic Ile108(B) | 1 x hbond Ile108(A) | 1 x hbond Gln115(B) |
| | 1 x hphobic Phe109(B) | 1 x hbond His110(B) | 1 x pistack Trp75(B) |
| | 1 x hphobic His110(A) | | |
| Isocarboxazid | 1 x hphobic Trp75(B) | 1 x hbond Lys77(B) | 2 x hbond Gln115(B) |
| | 1 x hphobic His110(B) | 1 x hbond Phe109(B) | 1 x pistack His110(A) |
| | 1 x hphobic Lys119(B) | | |
| Rilpivirine | 1 x hphobic Ile108(A) | 1 x hphobic His110(B) | 2 x hbond Gln115(B) |
| | 2 x hphobic Phe109(A) | 1 x hbond Ile108(B) | 1 x pistack His110(A) |
| Iloperidone | 1 x hphobic Trp75(B) | 1 x hphobic His110(B) | 1 x hbond Gln115(B) |
| | 1 x hphobic Ile108(A) | 2 x hphobic Gln115(B) | 1 x hbond Lys119(B) |
| | 2 x hphobic Phe109(A) | 1 x hphobic Lys119(B) | |
| Pralatrexate | 1 x hphobic Trp75(B) | 1 x hphobic Gln115(B) | 1 x hbond Gln115(B) |
| | 1 x hphobic Phe109(A) | 1 x hbond His110(A) | 1 x sbridge His110(B) |
| | 1 x hphobic His110(B) | 1 x hbond Glu111(A) | 1 x sbridge Lys119(A) |
| | 1 x hphobic Glu111(B) | 1 x hbond Gln115(A) | |

| | | | |
|---|---|---|---|
| Glycerol<br>phenylbutyrate | 2 x hphobic Trp75(B)<br>1 x hphobic Tyr81(B)<br>1 x hphobic Arg106(B)<br>3 x hphobic Ile108(A) | 1 x hphobic Phe109(A)<br>1 x hphobic His110(B)<br>1 x hphobic Glu111(B)<br>1 x hphobic Gln115(B) | 2 x sbridge His110(A)<br>1 x sbridge His110(B)<br>1 x pistack Trp75(B) |
| Bisacodyl | 1 x hphobic Trp75(B)<br>1 x hphobic Phe109(A)<br>2 x hphobic Phe109(B)<br>1 x hphobic His110(B) | 1 x hphobic Glu111(B)<br>1 x hphobic Gln115(B)<br>1 x hphobic Lys119(B)<br>1 x hbond Gln115(A) | 1 x hbond Gln115(B)<br>1 x sbridge Lys77(B)<br>1 x sbridge His110(B)<br>1 x sbridge Lys119(B |

**Table S3.** Top ten compound from virtual screening on TIM*Gl*.

| Compound | Structure | Docking score (Kcal/mol) | Description |
|---|---|---|---|
| Levocabastine | 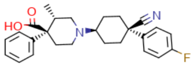 | -9.5 | Used for allergic conjunctivitis. |
| Etoposide | 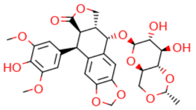 | -9.4 | Antitumor activity. |
| Sulfasalazine | 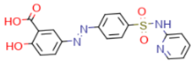 | -9.3 | Management of inflammatory bowel diseases. |
| Eltrombopag | 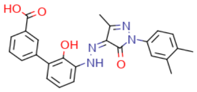 | -9.3 | Treatment of low blood platelet counts in adults with chronic immune thrombocytopenia. |
| Indinavir | 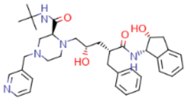 | -9.2 | Treatment of HIV-1 infections. |
| Difenoxin | 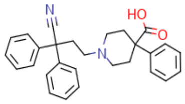 | -9.0 | Treatment of diarrhea. |
| Dolasetron | 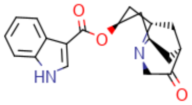 | -8.9 | Antinauseant and antiemetic agent. |
| Bosentan | 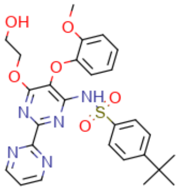 | -8.7 | Treatment of pulmonary hypertension. |
| Carvedilol | 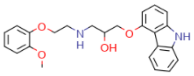 | -8.7 | Treatment of heart failure and hypertension. |

| | | | |
|---|---|---|---|
| Lapatinib | 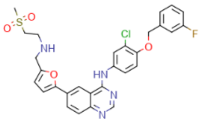 | -8.7 | Chemotherapy agent. |

**Table S4.** Interaction profile of top ten compound from virtual screening on TIM*Gl*.

| Compound | Interactions | | |
|---|---|---|---|
| Levocabastine | 1 x hphobic Tyr68(A) | 1 x hphobic Gln109(A) | 1 x hbond Lys113(B) |
| | 1 x hphobic Tyr68(B) | 1 x hphobic Gln109(B) | 1 x sbridge Arg99(A) |
| | 1 x hphobic Leu69(A) | 1 x hbond Glu105(B) | 1 x sbridge Lys113(A) |
| | 1 x hphobic Leu69(B) | | |
| Etoposide | 1 x hphobic Tyr68(A) | | 1 x hbond Gln109(B) |
| | 1 x hphobic Leu69(A) | 1 x hbond Glu78(B) | 1 x hbond Lys113(A) |
| | 1 x hphobic Gln109(A) | 1 x hbond Arg99(A) | 1 x sbridge Arg99(B) |
| | 1 x hbond Tyr68(A) | 1 x hbond Arg99(B) | 1 x sbridge Lys113(A) |
| | 1 x hbond Tyr68(B) | 1 x hbond Ile102(A) | 1 x sbridge Lys113(B) |
| Sulfasalazine | 1 x hphobic Tyr68(A) | 1 x hphobic Gln109(A) | 1 x hbond Arg99(B) |
| | 2 x hphobic Leu69(A) | 1 x hbond Tyr68(A) | 1 x hbond Met103(A) |
| | 1 x hphobic Met103(B) | 1 x hbond Glu70(A) | 1 x pication Arg99(B) |
| | 1 x hphobic Glu105(B) | 1 x hbond Arg99(A) | |
| Eltrombopag | 1 x hphobic Tyr68(A) | 1 x hphobic Glu105(B) | 1 x hbond Glu78(B) |
| | 1 x hphobic Leu69(A) | 1 x hphobic Gln109(A) | 1 x hbond Arg99(B) |
| | 2 x hphobic Leu69(B) | 1 x hphobic Gln109(B) | 1 x hbond Gln109(A) |
| | 1 x hphobic Met103(A) | 1 x hbond Tyr68(A) | 1 x pistack Tyr68(B) |
| | 1 x hphobic Glu105(A) | 1 x hbond Glu78(A) | 1 x pication Lys113(B) |
| Indinavir | 1 x hphobic Tyr68(B) | 1 x hphobic Glu105(B) | 1 x hbond Met103(A) |
| | 1 x hphobic Met103(B) | 1 x hbond Tyr68(B) | 1 x hbond Gln109(A) |
| | 1 x hphobic Glu105(A) | 1 x hbond Arg99(A) | 2 x hbond Gln109(B) |
| Difenoxin | 1 x hphobic Tyr68(A) | 1 x hphobic Gln109(B) | 1 x sbridge Glu78(B) |
| | 1 x hphobic Leu69(A) | 1 x hbond Arg99(B) | 1 x sbridge Arg99(B) |
| | 1 x hphobic Leu69(B) | 1 x hbond Met103(B) | 1 x sbridge Lys113(B) |
| | 1 x hphobic Glu105(A) | 1 x hbond Glu105(B) | 1 x pistack Tyr68(B) |
| | 2 x hphobic Gln109(A) | | |
| Dolasetron | 1 x hphobic Tyr68(A) | 2 x hphobic Glu105(B) | 1 x sbridge Arg99(A) |
| | 1 x hphobic Tyr68(B) | 1 x hbond Tyr68(A) | 1 x sbridge Arg99(B) |
| | 1 x hphobic Glu105(A) | 1 x hbond Arg99(B) | |
| Bosentan | 1 x hbond Glu78(B) | 1 x hbond Met103(B) | 1 x hbond Lys113(B) |
| | 2 x hbond Arg99(A) | 1 x hbond Glu105(B) | 1 x pistack Tyr68(A) |

| | | | |
|---|---|---|---|
| | 1 x hbond Arg99(B) | 1 x hbond Gln109(A) | |
| Carvedilol | 1 x hphobic Tyr68(A)<br>1 x hphobic Met103(A)<br>1 x hphobic Glu105(B)<br>1 x hbond Glu78(A) | 1 x hbond Arg99(A)<br>1 x hbond Arg99(B)<br>1 x hbond Gly104(A) | 2 x hbond Gln109(A)<br>1 x hbond Gln109(B)<br>1 x pication Arg99(A) |
| Lapatinib | 1 x hphobic Tyr68(A)<br>1 x hphobic Tyr68(B)<br>1 x hphobic Leu69(A) | 1 x hphobic Leu69(B)<br>1 x hphobic Glu105(B)<br>2 x hbond Arg99(B) | 1 x hbond Met103(A)<br>1 x hbond Glu105(B)<br>1 x hbond Lys113(B) |

**Table S5.** Top ten compounds selected from virtual screening against both TIM*Eh* and TIM*Gl*.

| Compound | Structure | Docking score (Kcal/mol) | Description |
|---|---|---|---|
| Clorhexidine | 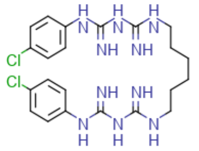 | -6.8[1]<br>-8.3[2]<br>-7.6[3] | Broad-spectrum antimicrobial. |
| Pemetrexed | 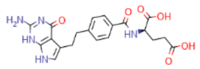 | -7.0<br>-8.0<br>-7.5 | Chemotherapy. |
| Dolasetron | 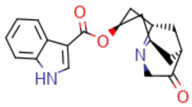 | -6.0<br>-8.9<br>-7.5 | Antinauseant and antiemetic agent. |
| Tolcapone | 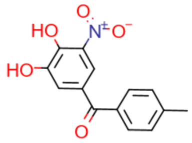 | -6.3<br>-8.5<br>-7.4 | Treatment of Parkinson´s disease. |
| Imatinib | 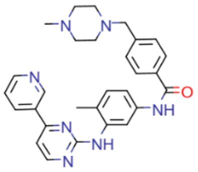 | -6.7<br>-8.1<br>-7.4 | Chemotherapy. |
| Arbutamine | 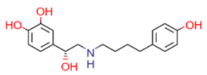 | -6.4<br>-8.3<br>-7.4 | Cardiac stimulant. |
| Folic acid | 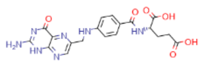 | -6.6<br>-7.9<br>-7.3 | Supplementation agent. |
| Isocarboxazid | | -6.8 | Treatment of major depression. |

| | | | |
|---|---|---|---|
| | 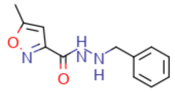 | -7.5<br>-7.2 | |
| Glycerol phenylbutyrate | 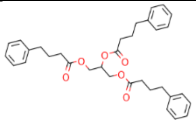 | -6.9<br>-7.5<br>-7.2 | Therapy for chronic management of patients with urea cycle disorders. |
| Phenindione | 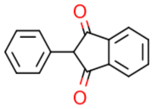 | -6.5<br>-7.7<br>-7.1 | Anticoagulant. |

[1] TIM*Eh* value
[2] TIM*Gl* value
[3] Mean value

**Table S6.** ADMET characteristics of selected compounds. Available at: https://go.drugbank.com/

| Compound | Absorption | Mechanism of action | Metabolism | Route of elimination | Toxicity |
|---|---|---|---|---|---|
| Chlorhexidine | Orally administered chlorhexidine is very poorly absorbed from the gastrointestinal. | Disrupt microbial cell membranes. | Unlikely to undergo metabolic conversion to any significant extent. | Exclusively via the feces. | The LD50 of subcutaneously administered chlorhexidine in mice is >5 g/kg. |
| Imatinib | Well absorbed with mean absolute bioavailability is 98% and maximum plasma levels achieved within 2-4 hours of dosing. | Inhibits the Bcr-Abl tyrosine kinase. | Primarily hepatic via CYP3A4. | 81% of the dose is eliminated within 7 days, 68% in feces and 13% urine. | The most frequently reported adverse reactions (>30%) were edema, nausea, vomiting, muscle cramps, musculoskeletal pain, diarrhea, rash, fatigue and abdominal pain. |
| Folic acid | Rapidly absorbed from the small intestine, primarily from the proximal portion. | Folic acid congeners are transported across cells by receptor-mediated endocytosis where they are needed to | Folic acid is metabolized in the liver into the cofactors dihydrofolate (DHF) and tetrahydrofolate (THF) by the | After a single oral dose of 100 mcg of folic acid in a limited number of normal adults, only a trace | IPR-MUS LD50 85 mg/kg<br>IVN-GPG LD50 120 mg/kg<br>IVN-MUS LD50 239 mg/kg<br>IVN-RAT LD50 500 mg/kg |

| | | | | | |
|---|---|---|---|---|---|
| | | maintain normal erythropoiesis, synthesize purine and thymidylate nucleic acids, interconvert amino acids, methylate tRNA, and generate and use formate. | enzyme dihydrofolate reductase (DHFR). | amount of the drug appeared in the urine. | IVN-RBT LD50 410 mg/kg |
| Tolcapone | Rapidly absorbed (absolute bioavailability is about 65%) | The precise mechanism of action of tolcapone is unknown, but it is believed to be related to its ability to inhibit COMT | The main metabolic pathway of tolcapone is glucuronidation | Almost completely metabolized prior to excretion, with only a very small amount (0.5% of dose) found unchanged in urine. | LD50 = 1600 mg/kg (Orally in rats) |

```
import concurrent.futures
import os
import time
import glob
import sys

molecules_path = sys.arg[1]
list_of_molecules = glob.glob('%s/*'%molecules_path)
config_file = sys.arg[2]

start = time.perf_counter()

def runVina(mol):
    print('Running %s...' % mol)
    out = mol.replace('ligands', 'out_vina')
    os.system('./vina --ligand %s --config %s --out %s' % (mol, config_file, out))

with concurrent.futures.ProcessPoolExecutor() as executor:
    results = [executor.submit(runVina, mol) for mol in list_of_molecules]

    for f in concurrent.futures.as_completed(results):
        print('Done...')
finish = time.perf_counter()
print('Finished in %f seconds' % (finish-start))
```

**Figure S1.** Python script for docking automatization
